# Neuroradiologischer, kinderradiologischer oder interventioneller Schwerpunkt – was für mich?

**DOI:** 10.1007/s00117-026-01593-8

**Published:** 2026-03-16

**Authors:** Andrea Jarre, Johanna Pape, Charlotte Wintergerst

**Affiliations:** 1 Institut für Diagnostisch und Interventionelle Radiologie und Neuroradiolgie, München Klinik Harlaching, Sanatoriumsplatz 2, 81545 München, Deutschland; 2 Institut für Kinderradiologie, Universitätsklinikum Leipzig, Liebigstraße 20A, 04103 Leipzig, Deutschland; 3 Klinik für diagnostische und interventionelle Radiologie, Universitätsklinikum Freiburg, Medizinische Fakultät, Albert-Ludwigs-Universität Freiburg, Hugstetter Str. 55, 79106 Freiburg, Deutschland

Die Radiologie zeichnet sich durch ein breites Spektrum aus. Neben vielfältigen diagnostischen Möglichkeiten spielen zunehmend therapeutische Verfahren eine große Rolle. Sie bildet dabei eine Schnittstelle der Medizin und begleitet von der Erstdiagnose über das Therapiemonitoring bis hin zur Therapie und Nachsorge. Radiolog*innen stehen im engen interdisziplinären Austausch mit nahezu allen Fachgebieten. Der rasche Ausbau der diagnostischen und therapeutischen Maßnahmen und kontinuierliche technische Weiterentwicklung sorgen für andauernden Wandel, bei dem man lebenslang dazu lernt.

## Radiologische Grundausbildung

**Abb. 1:**
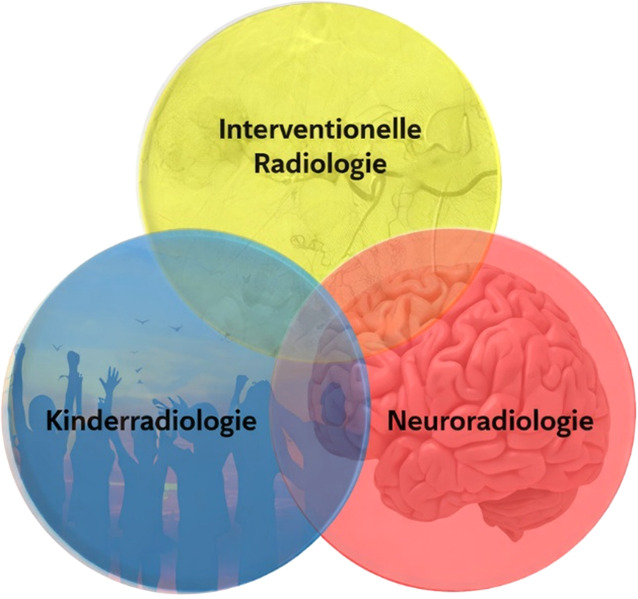
Schematische Darstellung der Überschneidungen der einzelnen Spezialisierungsmöglichkeiten in der Radiologie

Die Vielfalt und Masse der radiologischen Leistungen werden während der Facharztweiterbildung Radiologie deutlich. Die initiale Weiterbildung dauert 5 Jahre und kann je nach Weiterbildungsermächtigung in unterschiedlichen Kliniken und anteilig im niedergelassenen Sektor erworben werden. Doch wie geht es danach weiter? Neben dem Weiterarbeiten in der Allgemeinradiologie gibt es zahlreiche Möglichkeiten, sein Wissen und Fähigkeiten in einem Bereich zu erweitern und eine Spezialisierung anzustreben (Abb. 1). Dabei zeichnet die Radiologie aus, dass es ein solides Netzwerk gibt, mit diversen Möglichkeiten, sich auszutauschen, aktiv zu werden und die eigenen Interessen zu vertiefen. Initiativen wie das Forum Junge Radiologie [1], die Junge interventionelle Radiologie [2], die Junge Neuroradiologie [3] und die Junge Kinderradiologie [4] sind dabei essenzielle Säulen, die für einen Austausch bereits in frühen Ausbildungsjahren sorgen und eine gute Orientierungsmöglichkeit im Dschungel der Spezialisierungsmöglichkeiten bieten. Im folgenden Artikel werden aus dem breiten Spektrum einzelne Spezialisierungsmöglichkeiten exemplarisch aufgezeigt.

## Interventionelle Radiologie

Die Interventionelle Radiologie (IR) zeichnet sich dadurch aus, dass nicht die Diagnostik, sondern das therapeutische Handeln im Mittelpunkt steht; die Bildgebung wird zum Werkzeug für minimal-invasive Eingriffe. Unter Einsatz verschiedener bildgebender Modalitäten werden Katheter, Sonden, Nadeln und Drähte genutzt, um gezielte diagnostische oder therapeutische Maßnahmen an Patient*innen durchzuführen. Dabei bietet die IR ein facettenreiches Spektrum, das von minimal-invasiven onkologischen Therapien über gefäßverschließende und -eröffnende Eingriffe bis hin zu Schmerztherapien und vielem mehr reicht.

Der Alltag besteht aus einer spannenden Mischung aus elektiven Eingriffen und Notfallinterventionen sowie fortwährenden technischen Neuerungen. Die interdisziplinäre Zusammenarbeit, wie in Tumorboards und Fallbesprechungen, sowie Versorgung und Nachsorge von Patient*innen sind wichtige Bestandteile. Abgerundet wird das Spektrum durch die Teilnahme an interventionell-radiologischen Rufbereitschaften, die eine 24-stündige Patientenversorgung sicherstellen.

Aktuell besteht der Schwerpunkt IR in Deutschland noch nicht; allerdings gibt es Bestrebungen seitens der Deutschen Röntgengesellschaft (DRG) und der Deutschen Gesellschaft für Interventionelle Radiologie (DeGIR), dies zu ändern. Aktuell gibt es die Option der individuellen Qualitätssicherung über DeGIR-Zertifikate der Stufen 1 und 2 sowie über die Prüfung zum European Board of Interventional Radiology (EBIR). Nach abgeschlossener IR-Ausbildung besteht die Möglichkeit, in Kliniken verschiedener Versorgungsstufen und interdisziplinären Zentren zu arbeiten.

Die IR stellt hohe Anforderungen an die Ärzt*innen; neben einem breiten, aktuellen medizinischen Wissen und sehr guten Bildgebungskenntnissen zeichnen interventionell tätige Radiolog*innen auch ausgeprägt manuelle und technische Fertigkeiten sowie kognitive Kompetenzen aus, darunter Teamwork, Stressresistenz, Entscheidungsfreudigkeit und Kommunikationsstärke [5]. Trotz dieser hohen Anforderungen bildet die IR eine einzigartige Mischung aus Innovation, direkten Ergebnissen, Spannung, Teamwork und manuellem Arbeiten und eignet sich dementsprechend für alle, die genau dieses in ihrem täglichen Arbeitsalltag suchen.

## Kinderradiologie

Die Schwerpunktbezeichnung Kinderradiologie (KiR) umfasst die bildgebende Diagnostik bei Kindern und Jugendlichen einschließlich Sonographie, MRT, kindgerechte Röntgen- und CT-Diagnostik, strengen Strahlenschutz sowie die enge interdisziplinäre Zusammenarbeit mit pädiatrischen Fachdisziplinen, als auch den täglichen Austausch mit den jungen Patient*innen und Eltern.

Die KiR umfasst eine sehr kleine Fachgruppe aus aktuell ca. 150 tätigen Kinderradiolog*innen in Deutschland und profitiert durch einen sehr engen Austausch untereinander.

Die Schwerpunktbezeichnung wird nach abgeschlossener Facharztweiterbildung durch eine 24-monatige Weiterbildung an von den Landesärztekammern anerkannten Zentren erworben und qualifiziert vor allem für Tätigkeiten an universitären, ambulanten und spezialisierten pädiatrischen Einrichtungen; zusätzliche Zertifikate wie das European Diploma in Paediatric Radiology (EDiPR) und das European Diploma in Paediatric Neuroradiology (EDiPNR) können erworben werden.

Der Alltag in der KiR ist vielfältig, deutlich weniger planbar und erfordert mehr Flexibilität als in der allgemeinradiologischen Routine:

Geduld, Flexibilität und Kommunikation spielen eine ebenso große Rolle wie fachliche und technische Expertise. Untersuchungen dauern mitunter länger – manchmal auch mit Unterstützung eines Kuscheltiers – und verlangen ein gutes Gespür für unterschiedliche Altersstufen.

Fachlich prägen insbesondere Sonographie und MRT den Arbeitsalltag. Dabei begegnet man einem sehr breiten Spektrum an Krankheitsbildern, von häufigen Fragestellungen bis hin zu extrem seltenen Befunden, die man teilweise, auch mit jahrelanger Erfahrung, noch nie zuvor gesehen hat. Untersuchungsprotokolle geben Struktur, gleichzeitig müssen neue technische Entwicklungen und Strahlenschutz im Kindesalter besonders kritisch angepasst werden.

Ein wesentlicher Bestandteil der Arbeit ist der enge fachliche Austausch sowie die verständliche und einfühlsame Kommunikation mit Angehörigen und den kleinen Patient*innen selbst, um Vertrauen zu schaffen und eine hochwertige Diagnostik zu ermöglichen.

Die Weiterbildung zur Schwerpunktbezeichnung KiR ist anspruchsvoll, aber sehr bereichernd – für alle, die einen abwechslungsreichen Arbeitsalltag mit intensiver interdisziplinärer Kommunikation, engem Patienten- und Elternkontakt und kalkulierbarer Unplanbarkeit schätzen.

## Neuroradiologie

Die Neuroradiologie (NR) zeichnet sich durch ein vielfältiges und anspruchsvolles Spektrum aus, sowie einer breiten Palette an sich stets entwickelnder Diagnostik und Interventionen. Im Vordergrund steht die Zusammenarbeit im interdisziplinären Team, welche den klinischen Alltag maßgeblich prägt.

Die Zusatzweiterbildung NR umfasst 2 Jahre und kann an Universitätskliniken oder großen Schwerpunkthäusern mit Weiterbildungsermächtigung erfolgen. Kenntnisse können zusätzlich über spezielle DEGIR-Module (E + F) sowie den Erwerb von Zertifikaten der ESMINT (European Society of Minimally Invasive Neurological Therapy) vertieft werden.

Die interventionelle NR hat sich jüngst enorm weiterentwickelt, und perspektivisch ist von einer weiteren Zunahme der Interventionen auszugehen. Dies macht das Fach besonders zukunftsorientiert. Die NR eröffnet vielfältige berufliche Perspektiven in Klinik (Maximalversorger, Stroke Units und im Rahmen luftgebundener Rettungskonzepte), Niederlassung und Forschung.

Ein besonderer Reiz liegt im direkten Patientenkontakt: Neben der Akutversorgung können Patient*innen im weiteren Verlauf betreut und Therapieergebnisse verfolgt werden. Bei der Thrombektomie sind die Therapieeffekte oft eindrucksvoll, mit einem enormen unmittelbaren Nutzen für die Betroffenen mit resultierenden Erfolgserlebnissen.

Neben den vielen positiven Aspekten bringt das Fach auch Herausforderungen mit sich. Interventionelle Tätigkeiten erfordern ein hohes Maß an technischer Präzision, manueller Geschicklichkeit und Entscheidungsstärke. Zeitliche Flexibilität, Ruf- und Bereitschaftsdienste sowie körperlich und mental anspruchsvolle Interventionen gehören zum Alltag. Das Risiko von Komplikationen oder frustranen Interventionen kann psychisch belastend sein und setzt eine entsprechende Resilienz voraus.

Trotz – oder gerade wegen – dieser Anforderungen wird die NR von vielen als besonders erfüllendes Fach wahrgenommen. Die starke Gemeinschaft innerhalb des Fachs, das ausgeprägte interdisziplinäre Arbeiten, die enge Anbindung an klinische Partner und der oft unmittelbare und erhebliche Therapieerfolg machen die NR zu einem spannenden, hochrelevanten, wachsenden, vielseitigen und dynamischen Fachgebiet mit großer Zukunft.

## Fazit für die Praxis

Abschließend lässt sich sagen, dass dank des weiten Spektrums der Radiologie für alle etwas dabei ist.

Trotz der aufgezeigten Spezialisierungsmöglichkeiten eint alle Radiolog*innen das bildbasierte Arbeiten und der Status als Schnittstelle der Medizin. Erwähnenswert ist, dass zahlreiche Überschneidungen zwischen den aufgezeigten Schwerpunkten zu finden sind, wie z. B. die interventionelle Kinderradiologie, sodass trotz abgeschlossener Schwerpunktausbildung der Dialog innerhalb der radiologischen Gemeinschaft essenziell ist. Darüber hinaus gibt es viele weitere Spezialisierungsmöglichkeiten, wie die muskuloskeletale, gynäkologische oder kardiovaskuläre Radiologie. So vereint die Radiologie Spezialisierung, Austausch und Innovation in einzigartiger Weise.
